# Improvement on lipid production by *Scenedesmus obliquus* triggered by low dose exposure to nanoparticles

**DOI:** 10.1038/s41598-017-15667-0

**Published:** 2017-11-14

**Authors:** Meilin He, Yongquan Yan, Feng Pei, Mingzhu Wu, Temesgen Gebreluel, Shanmei Zou, Changhai Wang

**Affiliations:** Jiangsu Key Laboratory of Marine Biology, College of Resources and Environmental Science, Nanjing Agricultural University, Nanjing, 210095 China

## Abstract

Carbon nanotubes (CNTs), α-Fe_2_O_3_ nanoparticles (nano Fe_2_O_3_) and MgO nanoparticles (nano MgO) were evaluated for the effects on algae growth and lipid production. Nano Fe_2_O_3_ promoted cell growth in the range of 0–20 mg·L^−1^. CNTs, nano Fe_2_O_3_ and nano MgO inhibited cell growth of *Scenedesmus obliquus* at 10, 40 and 0.8 mg·L^−1^ respectively. Neutral lipid and total lipid content increased with the increasing concentration of all tested nanoparticles. The maximum lipid productivity of cultures exposed to CNTs, nano Fe_2_O_3_ and nano MgO was observed at 5 mg·L^−1^, 5 mg·L^−1^ and 40 mg·L^−1^, with the improvement by 8.9%, 39.6% and 18.5%. High dose exposure to nanoparticles limited increase in lipid productivity, possibly due to the repression on cell growth caused by nanoparticles-catalyzed reactive oxygen species (ROS) generation, finally leading to reduction in biomass and lipid production. Reduced accumulation of fatty acids of C18:3n3, C18:3n6 and C20:2 was observed in cells exposed to nanoparticles.

## Introduction

In recent days, diverse applications of nanoparticles (NPs) involves crop protection and production, cosmetics, drug delivery, photonic crystals, analysis, food, coatings, paints, catalysis and material science^1,2^. Exponentially increasing application of nanomaterials leads to the exposure of various nanoparticles in the aquatic environment, which attracts intense concerns regarding on their eco-toxicity risk assessment^3^. Metal nanoparticles, especially those containing heavy metal or trace metal ions, such as TiO_2_, ZnO, CuO, PbO, are found to be toxic to aquatic organisms^3–5^. The toxicity of nanoparticles to microalgae are known to be related to reactive oxygen species (ROS) generation inducing oxidative stress^3,6^, shading effect^7^ and agglomeration^8^. The disassociated ions from the NPs could impose a superposed toxic effect on aquatic organisms^9^. Noticeably, the responses of organisms to various NPs are both species and materials dependent. A few trials have been reported for the application of nanoparticles in improvement of crop production by enhancing germination, seedling growth and biomass production^10^, physiological activities including photosynthetic activity and nitrogen metabolism, and protein level (see more details in the review of^7^). Although many studies about the toxicity of oxide nanoparticles on microalgae have been reported^3–5^, only a few studies focused on the potential effects on algal biotechnology. Photopigments in *Chlorella vulgaris* were promoted by placing spheroidal silver nanoparticles and gold nanorods around microalgal culture flasks via such backscattering in the spectral regions favorable for microalgal growth^11^. Zero-valent iron nanoparticles were found to boost the growth of several green algae and eustigmatophycean algae at 5.1 mg·L^−1^ ^12^. The application of nanomaterials in algal biotechnology is still in its nascent phase and little is known about the experimental approaches and control of characteristics of the NPs to facilitate the development of NPs application in algae cultures. However, it should be noticed that highly concentrated nanoparticles embed with or uptake by algal biomass might induce public health risks if used in food or pharmaceuticals, while the risks are considered much lower when applied in algal biodiesel production.

Microalgae and their derived high value products have been extensively explored for commercial applications. Typically, algal lipid production attracts intense attention due to the increasing concern on biofuel production. Synthesis and accumulation of large amounts of TAGs, and the accompanying alterations in lipid and FA composition can occur in microalgae when exposed to oxidative stress imposed by chemical or physical environmental stimuli^13^. It’s assumed that typical nanoparticles, which induce oxidative stress, could be a candidate to promote algal growth and secondary metabolites accumulation, when applied at proper concentration through a proper way^14^. However, since NPs usually cause severe inhibition on cell growth, and impose strong oxidative stress resulting in cell damage and death at low concentrations, the oxidative stress induced by NPs usually exceeds the antioxidant defense of algae cells, thus putting the utilization of NPs under careful assessment and precise control to prevent oxidative damage to the cells.

The aim of this study is to test the positive effects of various nanoparticles on algal growth and accumulation of valuable biochemical products. Improvement on the biomass production by crop has been demonstrated by application of carbon nanotubes (CNTs)^10^, but little is known about the possible application of CNTs in microalgae, thus was selected as the first candidate material in this study. Two metal oxide NPs, hematite (α-Fe_2_O_3_) and magnesium oxide (MgO) were also selected considering their lower ecotoxicity^15,16^ and that the disassociated ions from the metal oxide NPs (Fe^3+^ and Mg^2+^) are essential to cell growth. Iron is essential to the metabolism and growth of all organisms and is especially important in phytoplankton because of its presence in iron–sulfur and cytochrome proteins involved in photosynthetic electron transport^17^. Appropriate elevated Fe concentration promotes cell growth and lipid production in *Chlorella sorokiniana* ^18^ and carbohydrates accumulation in *Dunaliella tertiolecta* ^19^. Magnesium is one of the key element required for chlorophyll synthesis and the co-factor for several important cellular processes^20^. Excessive Mg concentration and MgSO_4_ nanoparticles were found to enhance the lipid accumulation in *Chlorella vulgaris* cultivated in wastewater^21^.

The idea is to employ suitable nanoparticles, which are less toxic to organisms, as stimuli to alter cell metabolisms to boost accumulation of valuable biochemical compounds under controlled concentrations. Typical nanomaterials, CNTs, nano Fe_2_O_3_ and nano MgO were selected and their effects on biofuel production by a potential green algae *Scenedesmus obliquus* ^22^ were evaluated, with respect to cells growth, pigments, photosynthetic activity, soluble sugar and protein contents, and lipid production. Potential toxic evaluation of nanoparticles is also undertaken to explore a proper way to utilize nanoparticles in algal biotechnology.

## Results

### Effects of nanoparticles on cell growth of *S*. *obliquus*

**Figure 1:**
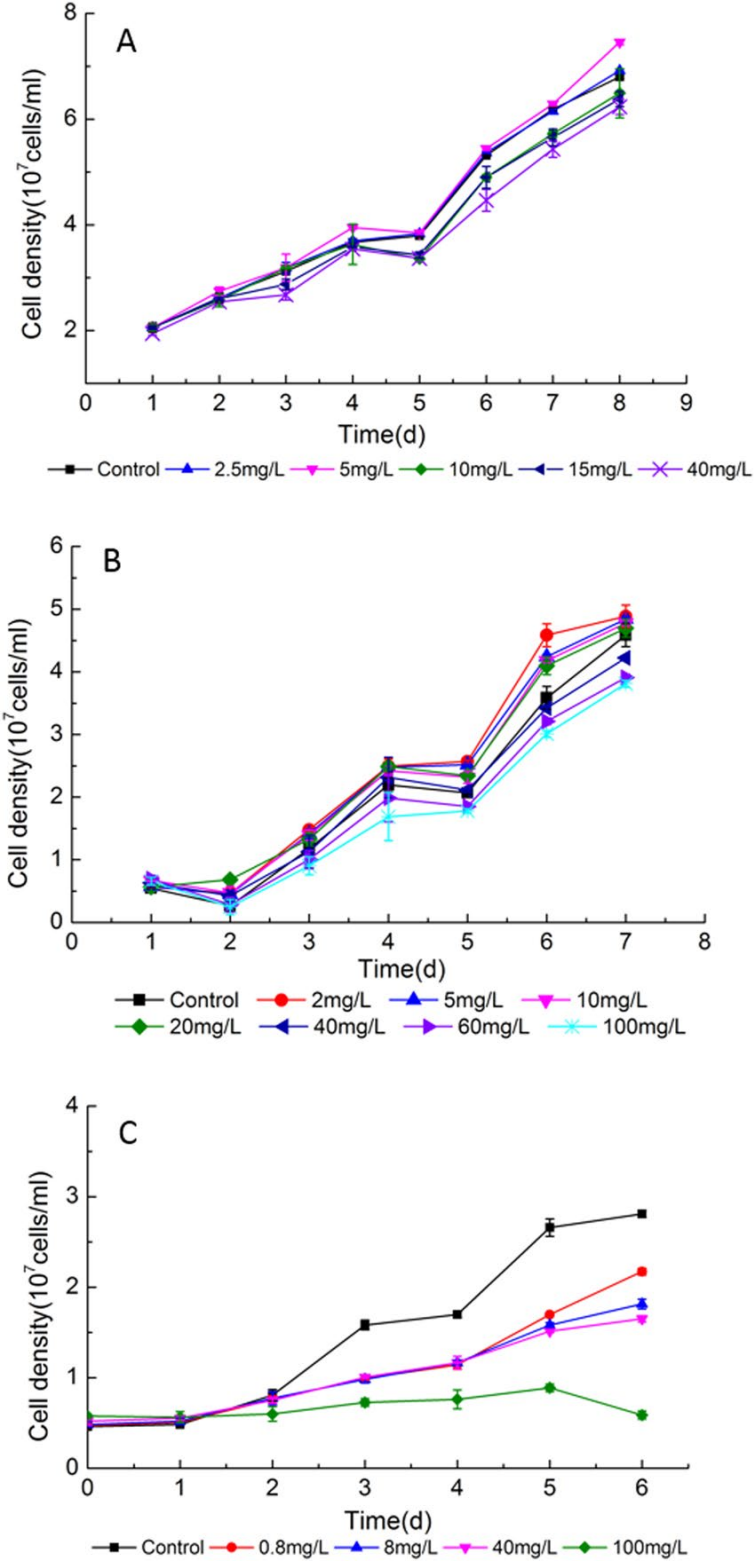
Growth curves of *S*. *obliquus* exposed to various concentrations of (**A**) CNTs, (**B**) nano Fe_2_O_3_ and (**C**) nano MgO.

Cells of *S*. *obliquus* grown under low concentrations of CNTs (2.5 and 5 mg·L^−1^) showed similar growth profiles as the untreated cells (Fig. 1A). High concentrations of CNTs (10, 15 and 40 mg·L^−1^) did not impose significant effect on cell growth within 96 h, but inhibited cell propagation after then. Promotion on cell growth was observed in cultures grown under tested nano Fe_2_O_3_ concentrations of 2–20 mg·L^−1^ (Fig. 1B). Concentrations higher than 40 mg·L^−1^ nano Fe_2_O_3_ led to slight inhibition on cell growth, with the reduction of 8.0%, 14.7% and 16.9% in the final cell density on 7 d at 40, 60 and 100 mg·L^−1^, respectively. Incubation of algae cells in medium containing nano MgO resulted in significant repression on cell growth even at lower concentration of 0.8 mg·L^−1^ at 72 h (Fig. 1C). The cell density was reduced by 22.7%, 35.4% and 41.1% under 0.8, 8 and 40 mg·L^−1^ nano MgO respectively. Increase of the nano MgO concentration to 100 mg·L^−1^ impeded *S*. *obliquus* propagation completely. EC_30_/EC_50_ calculated from the growth inhibition under treatment of CNTs and nano Fe_2_O_3_ was 59.62 mg·L^−1^ and 104.19 mg·L^−1^, 118.71 and 214.44 mg·L^−1^ respectively. EC_30_ and EC_50_ for nano MgO was 4.90 and 36.70 mg·L^−1^, which was much lower than that of CNTs and nano Fe_2_O_3_.

### Effects of nanoparticles on biochemical compounds

As shown in Table 1, a suitable amount of CNTs (5 mg·L^−1^) increased the chlorophyll content, while excessive CNTs repressed chlorophyll synthesis at 15 and 40 mg·L^−1^. Concentration of 2.5 and 10 mg·L^−1^ did not impose a significant effect. A similar increase in chlorophyll was observed in nano Fe_2_O_3_ treatment at 5, 10 and 20 mg·L^−1^, which was in agreement with the cell growth curves. Increasing concentration above 40 mg·L^−1^ nano Fe_2_O_3_ caused reduction in chlorophyll contents. Chlorophyll contents of cultures were greatly reduced by 73.3%, 84.5%, 80.6% and 87.5% when exposed to nano MgO of 0.8, 8, 40 and 100 mg·L^−1^, respectively. Protein contents were insignificantly different between treatments of various concentration of CNTs, while those in cultures exposed to nano Fe_2_O_3_ increased significantly at tested concentrations. Nano MgO induced noticeable increases in protein content at any tested concentrations, with the maximum protein content obtained at 100 mg·L^−1^. Increases in total soluble sugars were observed in all treatments with every tested NPs, resulting to the maximum content for CNTs, nano Fe_2_O_3_ and nano MgO at 15, 60 and 100 mg·L^−1^, respectively.

**Table 1:** Comparison of various physiological and biochemical parameters of *S*. *obliquus* exposed to CNTs, nano Fe_2_O_3_ and nano MgO.

	NPs (mg·L^−1^)	Chlorophyll (mg·L^−1^)	Chlorophyll (μg/10^8^ cells)	Protein (μg/10^7^ cells)	Soluble sugars (μg/10^7^ cells)	F_v_/F_m_	rETRmax
CNTs	0	5.52 ± 0.24	8.12 ± 0.35	1.29 ± 0.12	1.79 ± 0.14	0.595 ± 0.012	144.17 ± 1.12
	2.5	5.92 ± 0.45	8.56 ± 0.65	1.32 ± 0.25	2.32 ± 0.45*	0.605 ± 0.006	121.67 ± 6.94*
	5	6.92 ± 0.17*	9.28 ± 0.23*	1.36 ± 0.23	2.31 ± 0.27*	0.590 ± 0.014	134.33 ± 7.45
	10	5.80 ± 0.42	8.94 ± 0.65*	1.39 ± 0.20	2.16 ± 0.24*	0.530 ± 0.041*	114..34 ± 1.04*
	15	4.09 ± 0.39*	6.41 ± 0.61*	1.20 ± 0.17	2.60 ± 0.43*	0.333 ± 0.027*	70.78 ± 2.19*
	40	3.99 ± 0.19*	6.40 ± 0.30*	1.19 ± 0.07	2.01 ± 0.17*	0.351 ± 0.006*	87.53 ± 1.14*
Nano Fe_2_O_3_	0	4.66 ± 0.14	5.47 ± 0.11	1.57 ± 0.21	2.29 ± 0.12	0.570 ± 0.022	1517.33 ± 66.24
	2	4.75 ± 0.24	6.14 ± 0.18*	1.98 ± 0.11*	2.82 ± 0.22*	0.546 ± 0.036	1149.67 ± 103.94*
	5	5.67 ± 0.08*	6.15 ± 0.08*	1.72 ± 0.28	3.56 ± 0.43*	0.535 ± 0.064	1327.57 ± 94.47*
	10	5.18 ± 0.19*	5.88 ± 0.52	2.19 ± 0.32*	3.89 ± 0.17*	0.537 ± 0.020*	583.24 ± 81.05*
	20	4.87 ± 0.00*	5.13 ± 0.36	1.99 ± 0.23*	3.20 ± 0.25*	0.533 ± 0.047*	710.15 ± 21.13*
	40	4.47 ± 0.20	3.84 ± 0.09*	1.82 ± 0.03*	3.34 ± 0.37*	0.503 ± 0.005*	540.17 ± 12.14*
	60	4.30 ± 0.24*	3.76 ± 0.10*	1.92 ± 0.24*	4.04 ± 0.52*	0.492 ± 0.001*	368.67 ± 17.54*
	100	4.18 ± 0.26*	3.99 ± 0.17*	1.85 ± 0.09*	3.72 ± 0.65*	0.452 ± 0.001*	334.33 ± 56.50*
Nano MgO	0	4.39 ± 0.35	15.62 ± 1.25	3.67 ± 0.12	4.08 ± 0.05	0.555 ± 0.002	283.17 ± 11.30
	0.8	1.17 ± 0.16*	5.38 ± 0.74*	4.64 ± 0.08*	5.34 ± .03*	0.365 ± 0.006*	240.16 ± 5.36*
	8	0.68 ± 0.11*	3.75 ± 0.61*	5.21 ± 0.18*	5.06 ± 0.09*	0.390 ± 0.004*	210.34 ± 5.31*
	40	0.85 ± 0.12*	5.14 ± 0.73*	3.99 ± 0.20*	8.40 ± 0.17*	0.280 ± 0.001*	134.46 ± 3.28*
	100	0.55 ± 0.02*	9.36 ± 0.34*	8.14 ± 0.72*	12.52 ± 0.58*	0.133 ± 0.007*	44.88 ± 2.30*

Data are represented as mean ± SD from triplicate samples (*n = *3). Significant difference (*p* < 0.05) between treatments was indicated by asterisks.

### Photosynthetic parameters of *S*. *obliquus*

CNTs did not affect the maximum quantum efficiency F_v_/F_m_ values at concentrations lower than 10 mg·L^−1^, while it was remarkably repressed by 46.4–49.1% at concentrations of 15 and 40 mg·L^−1^ (Table 1). The values of the relative maximum electron transport rate (rETR_max_) of CNTs treatments showed consistent trend with their F_v_/F_m_ values. Low doses of nano Fe_2_O_3_ (2–20 mg·L^−1^) did not affect F_v_/F_m_, while high doses of nano Fe_2_O_3_ (40–100 mg·L^−1^) led to 11.7–20.7% of reduction. Noticeably, a remarkable inhibition on rETR_max_ (61.6%) was observed at 10 mg·L^−1^ of nano Fe_2_O_3_, while the F_v_/F_m_ was not greatly affected when compared with the untreated cultures. Under treatment of nano MgO, both F_v_/F_m_ and rETR_max_ decreased greatly with the increase in nano MgO concentrations.

### Lipid and fatty acids profiles

Lipid production parameters and fatty acids profiles of *S*. *obliquus* were determined under several representative NPs concentrations (Table 2). The neutral lipid fluorescence intensity was significantly promoted by increasing CNTs concentration. The total lipid content and lipid productivity was slightly promoted in cultures exposed to 5 and 40 mg·L^−1^ CNTs, compared to the untreated cells. The neutral lipid fluorescence gradually increased with increasing nano Fe_2_O_3_ concentrations, resulting a maximum value at 100 mg·L^−1^ of nano Fe_2_O_3_. Higher lipid content was observed in cultures under the exposure to 40 mg·L^−1^ and 100 mg·L^−1^ of nano Fe_2_O_3_, which was promoted by 27.8% and 44.8% when compared with the content in normal cultures. Nevertheless, the maximum lipid productivity was obtained at 5 mg·L^−1^ nano Fe_2_O_3_, with an increase by 39.6%. The lipid productivity of cultures treated with 40 and 100 mg·L^−1^ nano Fe_2_O_3_ was lower than that of 5 mg·L^−1^ but still higher than untreated cultures, probably due to the slight inhibition on biomass accumulation when the concentration of NPs increased from 5 mg·L^−1^ to 40 and 100 mg·L^−1^. Similar to the trend of nano Fe_2_O_3_, the neutral lipid fluorescence intensity of cells treated with nano MgO was greatly promoted with the increase in tested concentrations. A significant increase in total lipid content was observed in all treatments of nano MgO, which was 27.8% 32.2% and 53.4% higher than the control at the concentration of 0.8, 40 and 100 mg·L^−1^, respectively. A slight improvement in lipid productivity was observed in cultures treated with 0.8 mg·L^−1^ of nano MgO, while 100 mg·L^−1^ nano MgO resulted in lower lipid productivity, owing to the loss of biomass.

**Table 2:** Lipid production and fatty acid composition (%) of *S*. *obliquus* exposed to different concentrations of CNTs, nano Fe_2_O_3_ and nano MgO.

	CNTs			Nano Fe_2_O_3_				Nano MgO			
NPs concentration (mg·L^−1^)	0	5	40	0	5	40	100	0	0.8	40	100
Neutral lipid fluorescence (A.U/10^6^ cells)	4.55 ± 0.15	5.27 ± 0.15*	5.11 ± 0.34*	0.46 ± 0.01	0.56 ± 0.05*	0.81 ± 0.02*	1.40 ± 0.04*	1.36 ± 0.24	2.99 ± 0.22^*^	8.90 ± 0.24^*^	7.42 ± 1.06^*^
Total lipid content (% DCW)	22.94 ± 1.24	24.53 ± 0.33*	25.46 ± 0.77^*^	22.94 ± 1.24	25.14 ± 1.34^*^	29.24 ± 1.35^*^	33.22 ± 0.54^*^	22.94 ± 1.24	29.33 ± 1.11^*^	30.32 ± 0.99^*^	35.19 ± 1.31^*^
Lipid productivity (mg·L^−1^/d)	13.45 ± 0.14	14.65 ± 0.09^*^	14.21 ± 0.75^*^	13.45 ± 0.14	18.77 ± 0.07^*^	16.92 ± 0.16^*^	15.88 ± 0.22^*^	13.45 ± 0.14	15.94 ± 0.03^*^	15.03 ± 0.06^*^	11.58 ± 0.24^*^
C16:0	21.65 ± 0.41	22.92 ± 0.21*	23.38 ± 0.69*	21.65 ± 0.41	21.58 ± 0.35	22.98 ± 0.52*	22.66 ± 0.41*	21.65 ± 0.41	21.95 ± 0.09*	22.99 ± 0.97*	23.38 ± 0.31*
C16:1	2.18 ± 0.27	2.14 ± 0.11	2.85 ± 0.11*	2.18 ± 0.27	2.64 ± 0.23	2.45 ± 0.27	2.44 ± 0.31	2.18 ± 0.27	2.48 ± 0.72	2.84 ± 0.44*	5.35 ± 0.08*
C16:2	1.72 ± 0.06	2.45 ± 0.08*	3.29 ± 0.11*	1.72 ± 0.06	1.98 ± 0.41	3.29 ± 0.11*	5.08 ± 0.91*	1.72 ± 0.06	1.08 ± 0.11*	3.54 ± 0.12*	3.29 ± 0.24*
C18:0	3.46 ± 0.73	3.85 ± 0.27	3.49 ± 0.56	3.46 ± 0.73	3.96 ± 0.34	3.39 ± 0.63*	3.46 ± 0.19	3.46 ± 0.73	4.36 ± 0.31*	4.47 ± 0.22*	4.49 ± 0.41*
C18:1n9c	9.19 ± 0.33	10.73 ± 0.41*	10.23 ± 0.42*	9.19 ± 0.33	9.96 ± 0.42	10.06 ± 0.24	10.46 ± 1.01*	9.19 ± 0.33	9.46 ± 0.65	12.93 ± 1.23*	12.66 ± 0.56*
C18:2n6t	14.63 ± 0.97	9.29 ± 0.64*	10.49 ± 0.66*	14.63 ± 0.97	11.93 ± 0.18*	7.54 ± 0.86*	9.53 ± 2.00*	14.63 ± 0.97	10.26 ± 1.01*	5.37 ± 2.33*	7.49 ± 1.41*
C18:2n6c	8.12 ± 0.16	9.51 ± 0.18*	10.92 ± 0.86*	8.12 ± 0.16	9.81 ± 0.25*	12.66 ± 0.23*	10.11 ± 0.45*	8.12 ± 0.16	11.31 ± 0.99*	12.78 ± 0.55*	9.35 ± 0.72
C18:3n6	1.75 ± 0.22	1.86 ± 0.18	1.05 ± 0.08*	1.75 ± 0.22	1.49 ± 0.26*	0.99 ± 0.04*	1.69 ± 0.13	1.75 ± 0.22	1.86 ± 0.14	1.11 ± 0.20*	0.98 ± 0.10*
C18:3n3	31.14 ± 0.45	32.38 ± 0.75	29.29 ± 0.72	31.14 ± 0.45	30.77 ± 0.87	32.78 ± 0.53*	29.77 ± 1.22*	31.14 ± 0.45	32.77 ± 0.07*	28.45 ± 0.43*	26.7 ± 0.09*
C20:0	5.23 ± 0.29	3.94 ± 0.34*	4.33 ± 0.42*	5.23 ± 0.29	5.11 ± 0.24	2.31 ± 0.80*	4.12 ± 0.20*	5.23 ± 0.29	6.10 ± 0.34*	3.23 ± 0.20*	4.33 ± 0.39*
UFA/SFA	2.95 ± 0.25	2.70 ± 0.12	2.70 ± 0.12	2.95 ± 0.25	2.88 ± 0.11	2.72 ± 0.14	2.75 ± 0.15	2.95 ± 0.25	2.79 ± 0.16*	2.56 ± 0.11*	2.52 ± 0.48*

Fatty acid percentage was calculated as the ratios of the total fatty acids. UFA and SFA represent unsaturated and saturated fatty acids respectively.

Data are represented as mean ± SD from triplicate samples (*n = *3). Significant difference (*p* < 0.05) between treatments was indicated by asterisks.

Data for neutral lipid fluorescence were obtained from different biological repletion experiments, therefore the valves of the control groups of three tested NPs were different due to different cell density of independent experiments.

The contents of C16:0, C16:2, C18:1n9c and C18:2n6c were significantly increased by in treatments with low CNTs of concentration (5 mg·L^−1^), while those of C18:2n6t and C20:0 declined remarkably, and those of C16:1, C18:0, C18:3n6 and C18:3n3 were insignificantly affected (Table 1). The increases of C16:0, C16:2, C18:1n9c and C18:2n6c under 5 mg·L^−1^ CNTs were 5.9%, 42.4%, 16.8% and 17.1%, while the reduction in contents of C18:2n6t and C20:0 being 36.5% and 24.7% compared to the control. At 40 mg·L^−1^ CNTs, a more obvious increase in C16:1 by 30.7% and decrease in C18:3n6 by 40.0% was observed comparing to untreated cells. The contents of C16:0, C16:2, C18:1n9c and C18:2n6c were elevated with the increasing concentrations of nano Fe_2_O_3_. Reduction of fatty acid portion was determined in C18:2n6t, C18:3n3 and C20:0 in cultures exposed to increasing nano Fe_2_O_3_ concentrations. Significant reduction in C18:3n6 was only observed at a concentration of 5 and 40 mg·L^−1^ of nano Fe_2_O_3_. The contents of C16:0, C16:1, C16:2, C18:0, C18:1n9c increased with the increasing concentrations of nano MgO when it was higher than 0.8 mg·L^−1^, although reduced contents of C18:2n6t, C18:3n6, C20:0 were detected at the same concentration range. At 0.8 mg·L^−1^ nano MgO did not significantly affect the C16:0, C16:1, C18:0, C18:1n9c and C18:3n6. Only low dose of MgO (0.8 mg·L^−1^) induced elevation in C18:2n6c (which was also increased at 40 mg·L^−1^), C18:3n3 and C20:2 contents. The unsaturated fatty acid/saturated fatty acid (UFA/SFA) ratio declined negligibly under high concentrations of CNTs (40 mg·L^−1^) and nano Fe_2_O_3_ (40 and 100 mg·L^−1^). Low concentration of 0.8 mg·L^−1^ nano MgO caused a decrease in UFA/SFA ratio, which was further decreased with the increasing nano MgO concentrations.

### NPs induced oxidative stress and cellular antioxidant defenses

**Figure 2:**
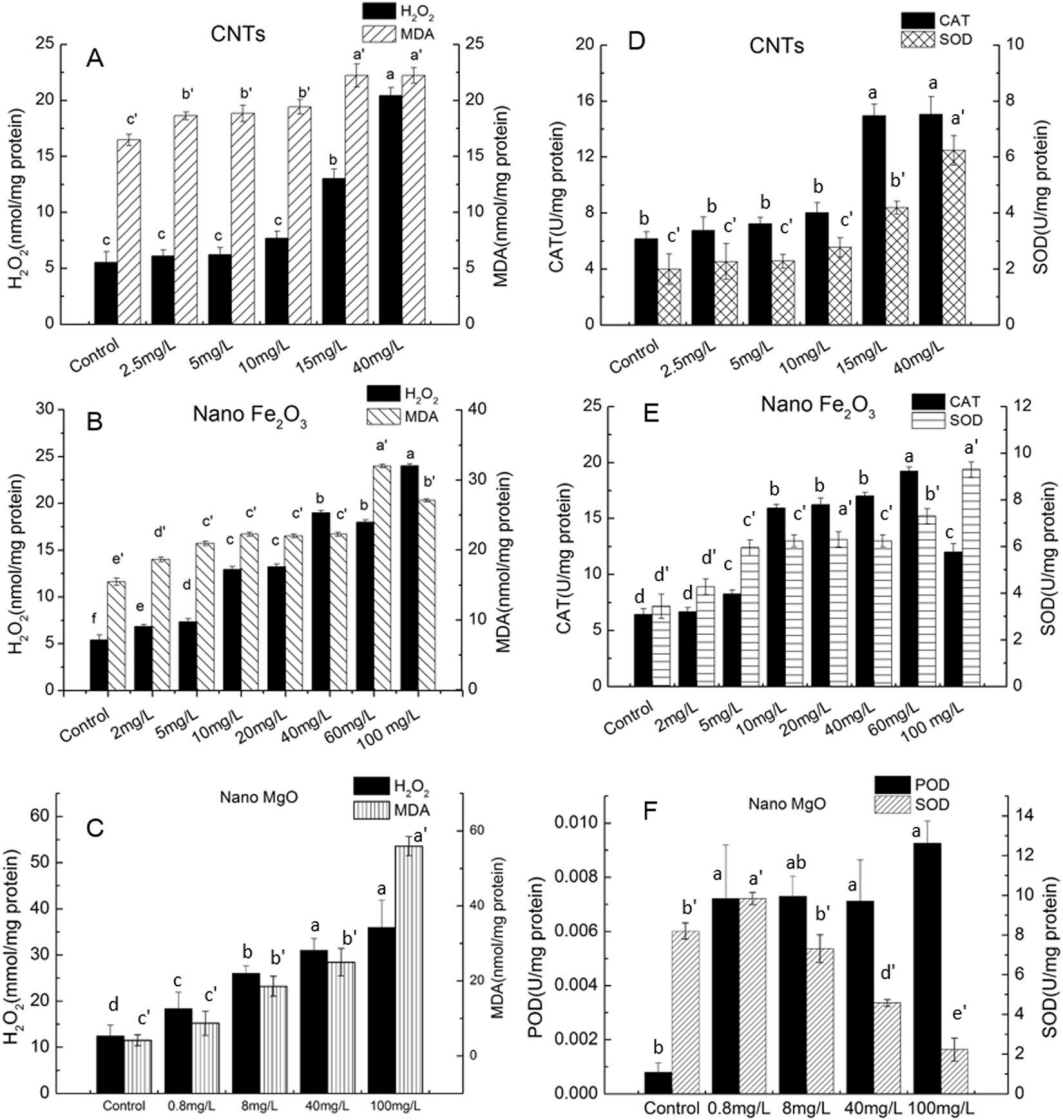
H_2_O_2_ and MDA contents of *S*. *obliquus* exposed to (**A**) CNTs, (**B**) nano Fe_2_O_3_ and (**C**) nano MgO for 48 h. Antioxidants enzyme activities (CAT, SOD or POD) of *S*. *obliquus* exposed to (**D**) CNTs, (**E**) nano Fe_2_O_3_ and (**F**) nano MgO for 48 h. Significant difference (*p* < 0.05) is indicated by different letters above the columns between treatments.

**Figure 3:**
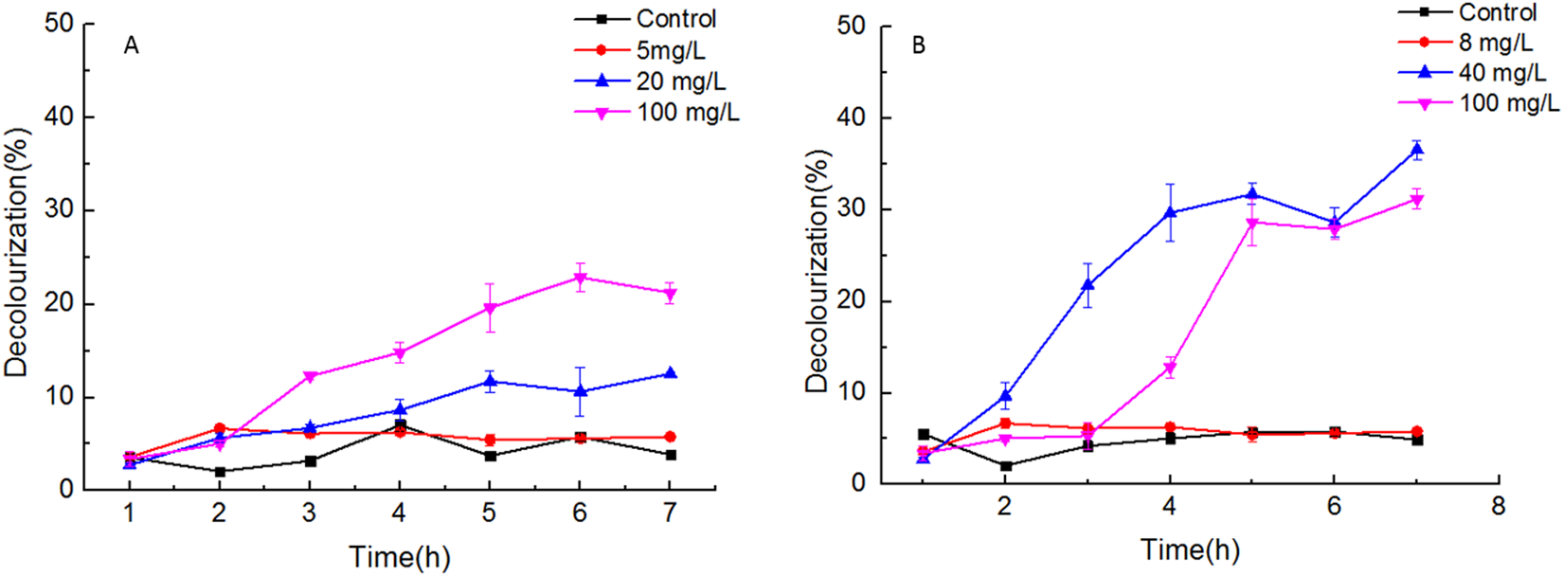
Decolourization curves of methylene blue in medium containing various concentrations of (**A**) nano Fe_2_O_3_ and (**B**) nano MgO.

To evaluate the oxidative stress induced by NPs, hydrogen peroxide, an important marker of ROS, was determined after 48 h of exposure to NPs. Although, insignificant differences in H_2_O_2_ contents of treatments exposed to low doses of CNTs (2.5, 5 and 10 mg·L^−1^) were observed (Fig. 2A), the H_2_O_2_ content was elevated at 15 mg·L^−1^ and 40 mg·L^−1^ CNTs treatment. Unlike CNTs, the H_2_O_2_ contents in cultures exposed to nano Fe_2_O_3_ (Fig. 2B) and nano MgO (Fig. 2C) increased with the increase in NPs concentrations. Furthermore, malondialdehyde (MDA) contents of cultures after incubation in NPs treatments for 48 h were measured to evaluate its potential oxidative damage to cell membrane. The MDA contents in treatments with CNTs, nano Fe_2_O_3_ and nano MgO increased to different extents, compared to the untreated cells. Methylene blue (MB) degradation curves were monitored to verify the photocatalytic ROS generation by nano Fe_2_O_3_ and nano MgO in cell-free medium under the same growth conditions that all experiments were carried out (Fig. 3). The results revealed that in the absence of nano Fe_2_O_3_ and nano MgO, MB was not degraded efficiently, indicating no ROS generation without addition of nano Fe_2_O_3_ or nano MgO. Low doses of nano Fe_2_O_3_ (5 mg·L^−1^) and nano MgO (8 mg·L^−1^) did not result in efficient MB degradation. As expected, the MB was degraded in the presence of nano Fe_2_O_3_ and nano MgO at high concentrations. MB cannot be completely degraded under 20 and 100 mg·L^−1^ of nano Fe_2_O_3_, with the decolorization ratio of 12.6% and 21.2% after 7 h incubation (Fig. 3A). The MB was rapidly decolorized within seven hours at 40 and 100 mg·L^−1^ MgO, with a decolorizing ratio of 36.6% and 31.7%, respectively (Fig. 3B).

As presented in Fig. 2D, the catalase (CAT) and superoxide dismutase (SOD) activities were greatly enhanced under 15 and 40 mg·L^−1^ CNTs. A substantial enhancement on the CAT activities of cells exposed to nano Fe_2_O_3_ was observed when the concentration increased from 10 to 60 mg·L^−1^ (Fig. 2E). The CAT activities decreased when the nano Fe_2_O_3_ was further increased to 100 mg·L^−1^, which was still higher than the control. The SOD activity of cells exposed to nano Fe_2_O_3_ increased greatly at concentrations higher than 10 mg·L^−1^, which maintained a relatively higher activity at 100 mg·L^−1^. The peroxidase (POD) activities of treatments with nano MgO (Fig. 2F) showed a remarkable enhancement at very low concentration (0.8 mg·L^−1^). The POD activities among the treatments with various nano MgO concentrations showed negligible differences between treatments. Unlike CNTs and nano Fe_2_O_3_, nano MgO triggered an increase in SOD activities at 0.8 mg·L^−1^, which then declined with the increasing nano MgO concentrations (Fig. 2F).

### SEM images

**Figure 4:**
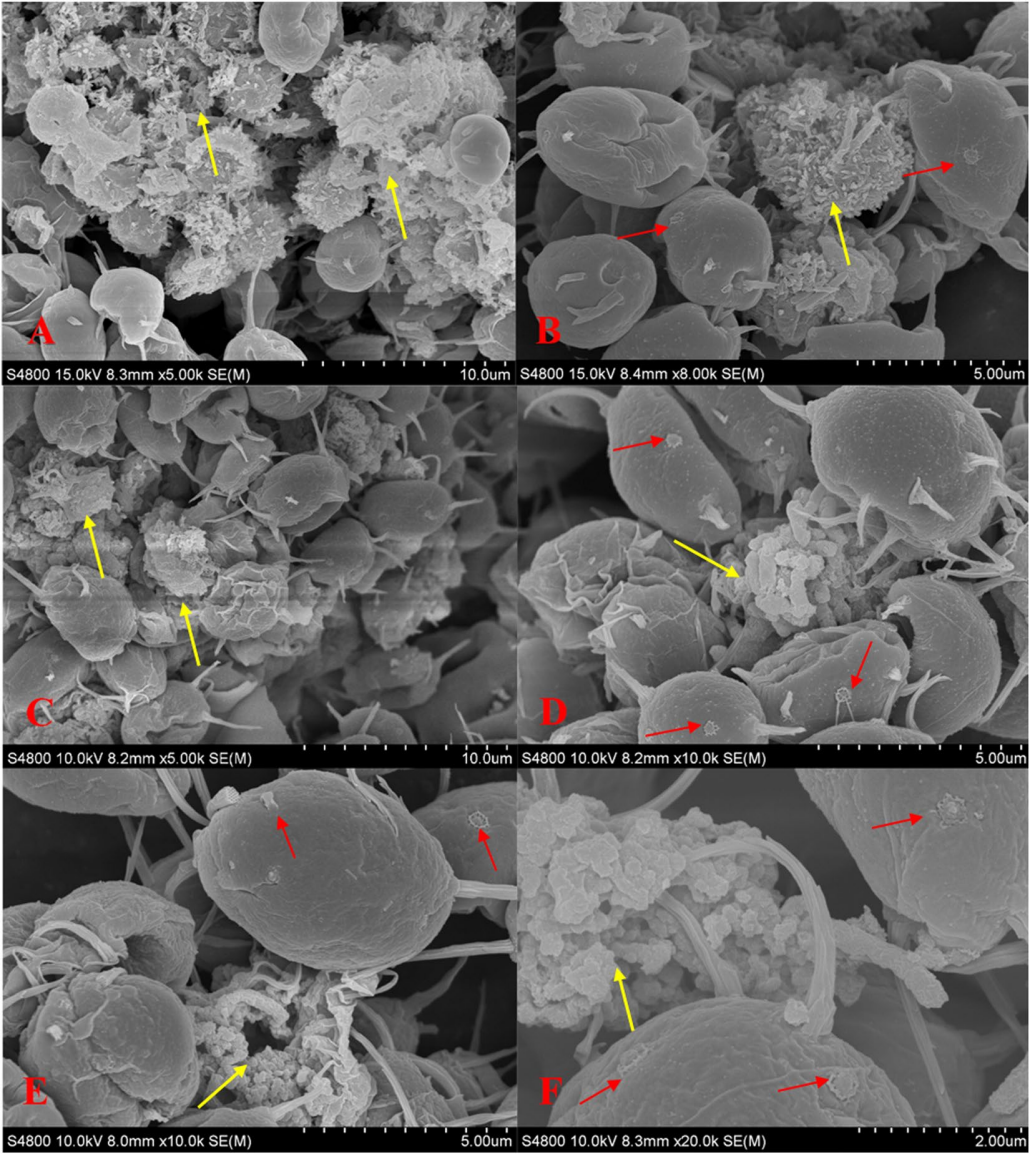
SEM images of *S*. *obliquus* exposed to (**A**) and (**B**) 40 mg·L^−1^ CNTs, (**C**) and (**D**) 60 mg·L^−1^ nano Fe_2_O_3_, (**E**) and (**F**) 40 mg·L^−1^ nano MgO for 96 h. Yellow arrows indicate the aggregates formed on by nanoparticles. Red arrows indicate the fracture sections of detached flagellums left on cell surface.

SEM images (Fig. 4) showed that shrank and distorted cells were observed in treatments of CNTs, nano Fe_2_O_3_ and nano MgO at tested concentrations. High concentrations of CNTs, nano Fe_2_O_3_ and nano MgO formed aggregates coating the cell surface, leading to the super aggregates formed by cells and nanoparticles after 96 h cultivation. Noticeably, all tested nanoparticles bound to the flagellums of *S*. *obliquus* and entrapped the flagellums into the NPs-aggregates. Random breakage of flagellums occurred more frequently, resulting in the detachment of flagellums from cell surface, as clearly indicated by the visible fracture spots left on the cell surface (Red arrows in Fig. 4).

### Dissociated Fe^3+^ concentration

**Figure 5:**
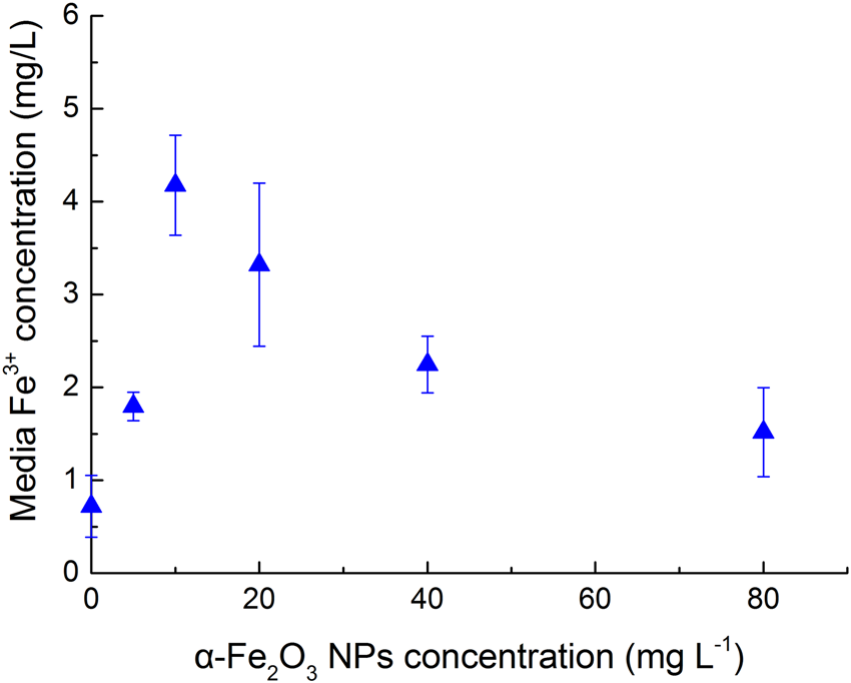
Fe^3+^ concentration in the culture exposed to different doses of nano Fe_2_O_3_.

As depicted in Fig. 5, the Fe^3+^ dissociated by nano Fe_2_O_3_ increased with increasing nano Fe_2_O_3_ concentration, when the NPs concentration was lower than 10 mg·L^−1^. The maximal dissociated Fe^3+^ concentration at 10 mg·L^−1^ was about 4.8 times higher than that of the control (BG11 medium). The dissociated Fe^3+^ strength decreased as the nano Fe_2_O_3_ concentration kept continually increasing. The Fe^3+^ concentration at 5 mg·L^−1^ of nano Fe_2_O_3_ showed insignificant difference with that at 80 mg·L^−1^ of nano Fe_2_O_3_.

## Discussion

Algal growth assays demonstrated CNTs, nano Fe_2_O_3_, and nano MgO inhibited cells growth of *S*. *obliquus* to different extents under high concentrations. The cell growth inhibiting concentration of CNTs and nano Fe_2_O_3_ was 10 and 40 mg·L^−1^, respectively. However, tested concentrations of CNTs and nano Fe_2_O_3_ did not induce substantial inhibition on cell growth, with the maximum inhibition effect of 22.7% and 23.1% at 40 and 100 mg·L^−1^, respectively. On the contrary, low concentration of nano MgO (0.8 mg·L^−1^) could repress cell growth significantly by 45.8% (Fig. 1C), indicating a higher toxic effect on cell growth and vitality than CNTs and nano Fe_2_O_3_ in this study. The lower EC_50_ value of nano MgO than CNTs and nano Fe_2_O_3_ also supported this result. Lower MB decolourization efficiency of nano Fe_2_O_3_ compared with nano MgO indicates less ROS generation in nano Fe_2_O_3_ cell-free solution, which might be one of the possible reasons for lower toxicity of nano Fe_2_O_3_ than nano MgO. Substantial decreases in F_v_/F_m_ of algal cells exposed to tested nanoparticles at inhibiting concentrations indicated that the photochemical activity was repressed and occurrence of photoinhibtion was inevitable^23^. Simultaneous reduction in rETR_max_ suggested the block of photosynthetic electron transport^24^. Several studies reported for the toxicity of CNTs to green algae. Schwab *et al*.^7^ found the inhibition on algae cell growth by aggregates of CNTs was highly correlated with the shading of CNTs and the agglomeration of algal cells, suggesting that the reduced algal growth might be caused mainly by indirect effects like limited light availability. Demir *et al*.^25^ reported the toxicity of α-Fe_2_O_3_ and γ-Fe_2_O_3_ NPs on *Nannochloropsis* sp. and *Isochrysis* sp. appeared at a low concentration of 1 mg·L^−1^. An opposite observation of higher toxicity of α-Fe_2_O_3_ than γ-Fe_2_O_3_ to *Chlorella pyrenonedosa* was reported in Lei’s work^15^, with the IC_50_ of ~ 71.0 mg·L^−1^ and ~132.0 mg·L^−1^, respectively. The much higher EC_50_ results in this study than the aforementioned researches, indicated a lower toxic effect of tested nano Fe_2_O_3_ to *S*. *obliquus*. Differences in size, periodical vacancies, crystal structure and opacity in suspension of nanoparticles are considered to be key factors controlling the toxicity of NPs^25^. Aruoja *et al*.^16^ reported the nontoxic effect of MgO on a green alga *Pseudokirchneriella subcapitata* and three other bacterial with an EC_50_ > 100 mg·L^−1^. By contrast, nano MgO was found to be toxic to *S*. *obliquus* even at low concentration in this study. The difference of sensitivity to nano MgO might be species-dependent. In general, the EC_50_ of CNTs (104.19 mg·L^−1^) and nano Fe_2_O_3_ (214.44 mg·L^−1^) are much higher than that of nano ZnO, CuO and nano TiO_2_, indicating lower environmental risks in application^4^.

The common toxicity mechanism of the NPs involves the generation of ROS, leading to oxidative damage to protein and other macromolecules with an ultimate cellular organelles injury^9,26^. Upon oxidative conditions, antioxidant defense mechanisms employed by algae include activation of reactive oxygen scavenging enzymes, such as CAT, SOD, POD, and production of the antioxidants including carotenoids and ascorbic, proteins and sugars^27^. The balance between ROS level and antioxidant defense capacities determines the occurrence of oxidative stress response and cell damage degree^6,27^. Remarkable increases in ROS levels and increased MDA contents (Fig. 3A–C), which is an important indicator for lipid peroxidation under oxidative stress^27^, were observed in cultures exposed to all tested NPs at cell growth repressing concentrations repressing cell growth. Although amounts of protein become the targets of ROS, oxidative stress could induce protein accumulation at low ROS level. The observation of increase in soluble protein content is usually considered as an evidence of active defense mechanism to prevent algae cells from damaging by abiotic stress^28^. The important components of soluble protein, antioxidant^29^ and biotransformation enzymes^30^, which are involved in the active defense mechanisms, are usually regarded as biomarkers for identification for xenobiotics. In addition, the elevation in protein content could be related to heat shock protein formation^31^ and elevation of specific mitochondria enzymes, such as alternative oxidase and uncoupling proteins^30^ in forestalling ROS production. Polak N reported that metallproteins and phytochelatin synthase may play an important role in response to nanoparticles^32^. The protective effects of soluble sugars against oxidative stress have been mostly attributed to signaling, triggering the production of specific ROS scavengers, and even acting as direct ROS scavengers at higher concentrations^33^. Glucose and sucrose play a central role in ROS signaling^33^. The longer water-soluble oligo- and polysaccharides might be effective candidates for capturing ROS and scavenging the free radicals in microalgal cells exposed to a wide range of environmental stresses^34^. The enhancement in the synthesis of sugars might represent another protective mechanism of environmental stress^35^, which is in accordance with the increase of cellular soluble sugars exposed to NPs in this study.

In a word, the enhancement on CAT, SOD or POD, and increases in soluble proteins and sugars of cultures exposed to nano Fe_2_O_3_ and nano MgO signify the involvement of both antioxidant enzymes and biochemical compounds in the antioxidant defense against the oxidative stress of NPs. When the oxidative stress induced by NPs exceeds the scavenging capacity of the cellular antioxidant defenses, oxidative damage is occurred finally leading to cell growth inhibition. In addition, large aggregates formed by NPs on cell surfaces might cause a shading effect on cell growth at high NPs concentrations by reducing the light availability^7^. The loss of the flagellums of *S*. *obliquus* and agglomerating with the NPs (Fig. 4), leading to serious limitation of cell motility, could also contribute to the toxicity at high NPs concentrations.

Interestingly, nano Fe_2_O_3_ at a concentration < 20 mg·L^−1^ promoted cell growth of *S*. *obliquus* (Fig. 1B), as well as the chlorophyll, soluble protein and sugar contents (Table 1). However, photosynthetic parameters were not enhanced under such conditions. Nano Fe_2_O_3_ at a concentration of 10 mg·L^−1^ induced oxidative stress while inhibition on cell growth occurred at a concentration higher than 40 mg·L^−1^. This indicates that the cells could somehow overcome the oxidative stress and maintain stimuli on cell growth in the range of 0–20 mg·L^−1^ nano Fe_2_O_3_. This promotion on algae growth might be related to the disassociated trace ions of nanoparticles (Fig. 5), which represents a suitable source of trace elements^36^. The dissociated Fe^3+^ from nano Fe_2_O_3_ especially at low concentration (<10 mg·L^−1^), might impose a positive effect on cell growth and biochemical compounds accumulation. Similar promotion on algae growth was reported by Pádrová *et al*.^12^; the trace concentrations of zero-valent iron nanoparticles (5.1 mg·L^−1^) caused overproduction of biomass during cultivation of cyanobacteria and microalgae. At high NPs concentrations, algal cell growth was repressed due to the NPs-induced oxidative injury. The promoting effect imposed by dissociated Fe^3+^ might be eliminated by the inhibition on cell growth, when Fe_2_O_3_ NPs concentration increased from low (5 mg·L^−1^) to high concentration (>40 mg·L^−1^). In summary, nano Fe_2_O_3_ imposed a stimulating effect on cell growth, while high concentrations of tested NPs imposed inhibition, or even toxicity (100 mg·L^−1^ nano MgO).

Majority of microalgae accumulate neutral lipids up to 20–50% of dry cell weight as a storage energy source under photo-oxidative stress or other adverse environmental conditions^13^. In this trial to explore the potential of CNTs, it was found they impose a positive effect at low concentration (5 mg·L^−1^) on neutral lipid accumulation (Table 2). However, the neutral lipid accumulation was not significantly improved when the CNTs concentration increased from 5 mg·L^−1^ to 40 mg·L^−1^ (*p* = 0.268). The increase in total lipid content at 40 mg·L^−1^ CNTs might be resulted from the increase in other types of lipids such as glycol lipids (GLs) and phospholipids (PLs), which are important components of external and chloroplast membrane, along with the endoplasmic reticulum^37^.

On the contrary, increasing concentrations of nano Fe_2_O_3_ and nano MgO greatly promoted the neutral lipids accumulation (Table 2). The optimal concentration of nano Fe_2_O_3_ and nano MgO for lipid productivity was 5 and 0.8 mg·L^−1^, respectively. Further increase of NPs concentration could not improve the lipid productivity. This could possibly be due to the less biomass accumulation as a result of repression on cell growth. The optimum concentration of ferric ions for biomass and lipid accumulation in green algae, such as *Chlorella vulgaris*, *Tetraselmis subcordiformis*, *Nannochloropsis oculata* and *Pavlova viridis*, was studied^38,39^. Very low Fe^3+^ concentration (0.0012 μM–1.2 μM) promoted cell growth, but only high levels of Fe^3+^ > 12 μM greatly enhanced both biomass and lipid production. Supplement of high concentrations of Fe could also increase the proportion of saturated fatty acids, which meets the requirements of qualified biodiesel production^39^.

As an essential element of chlorophylls, the beneficial effect of magnesium on biomass and lipid production by microalgae has been reported in mixotrophic conditions^40^. Saurabh *et al*. synthesized MgSO_4_ nanoparticles with sizes ~100 nm was found to greatly promote both biomass and lipid production at 1 g/L using crude glycerol as a substrate^21^. Nanoparticles of Mg aminoclay was reported to improve the lipid productivity of a mixotrophically grown *Chlorella* sp. by ~25%^41^. On the contrary to the above results obtained under mixotrophic growth condition, our results showed that the nano MgO can not promote both biomass and lipid production under photoautotrophic conditions. Further work could be carried out to overcome the limitation on cell growth by nano MgO, possibly under mixotrophic growth condition.

Comparing the fatty acid profiles of cells exposed to different nanoparticles with untreated cells, a common increase in C16:0, C16:1, C16:2, C18:1n9 and C18:2nc and decrease in contents of C18:2nt and C18:3n6 and C18:3n3, C20:2 was observed in treatments exposed to high concentrations of NPs. The UFA/SFA ratios declined slightly under high concentrations of NPs. The converse observation of decreased UFA contents in this study was against the reports of the accumulation of polyunsaturated fatty acids (PUFAs)^12,14^, which is known to have a radical scavenging potential and contribute to cell protection against increased ROS level^42^. One possible explanation is that the efficient involvement of UFAs in ROS scavenging might result in a metabolic interconversion between different FAs. Reduced accumulation of PUFAs such as C18:3n3, C18:3n6 and C20:2 in cells exposed to NPs is beneficial for controlling the oxidation stability of the downstream biodiesel products.

Although the improvement on lipid production by the tested NPs was not as effective as other environmental factors such as nutrients deprivation and UV treatment, the results are still promising that the tested NPs could improve lipid productivity at lower concentrations. Despite the fact that CNTs, nano Fe_2_O_3_ and nano MgO repressed cell growth at high concentrations, which might be related to ROS generation and agglomeration of algae cells and NPs, *S*. *obliquus* was able to overcome those adverse oxidative stresses typically at lower doses of NPs and diverted the metabolisms to lipid accumulation. Although the improvements achieved are still not soaring, nanoparticles can be considered as an auxiliary tool for enhancing lipid production by combining with other stimuli. Further improvements could be focused on combining various environmental stimuli with NPs to improve lipid production. Another advantage of the application of nanoparticles in algal biotechnology relates to the agglomeration of magnetic NPs and algae cells, facilitating the precipitation of algae cells from the medium, which is economically beneficial for cell harvesting. With respect to lipid extraction process, aminoclay-based nanoparticles, such as Fe-(3-aminopropyl)-triethoxysilane (Fe-APTES) clay imposed positive effects on lipid extraction yield, FAME content and its productivity of *Chlorella* sp. KR-1, through destabilization of algal cell walls via solubilized aminoclays and disruption in cell walls by hydroxyl radicals (OH·) originated from the Fenton-like reaction induced by Fe^3+^ ^43^.

In summary, nano Fe_2_O_3_ is found to be a promising candidate for improving both biomass and lipid production by *S*. *obliquus* at an appropriate concentration (<20 mg·L^−1^). Further increasing nano Fe_2_O_3_ concentrations caused gradual inhibition on cell growth, but still can facilitate lipid accumulation without limiting the lipid productivity. CNTs and nano MgO imposed a more severe repression on cell growth than nano Fe_2_O_3_. Exposure of *S*. *obliquus* to low doses of CNTs (5 mg·L^−1^) and nano MgO (0.8 mg·L^−1^) can promote lipid productivity while the lipid productivity was limited by reduction in biomass accumulation under high dose exposure.

## Materials and Methods

### Nanoparticles

Carbon nanotubes (CNTs, purity > 90%, diameter < 2 nm, length < 20 μm) was provided by Nanjing University of Science and Technology. Nano ferric oxide (α-Fe_2_O_3_, purity > 99.5%, particle size < 30 nm), nano MgO (purity > 99%, particle size < 50 nm), which was purchased from Aladdin Chemistry Co. Ltd, China, was used to prepare nanoparticle suspensions. Prior to every assay, fresh NPs stock suspensions (100 mg·L^−1^) were prepared by sonication for 60 min and then filtering through 0.22 μm pore size PC membranes (Millipore). Transmission electron microscopy images of nanoparticles were provided in the supplement materials.

### Strains and culture conditions

*Scenedesmus obliquus* was obtained from the Freshwater Algae Culture Collection, Institute of Hydrobiology, Chinese Academy of Sciences. The strain was pre-cultured aseptically in 250 mL Erlenmeyer flasks with 100 mL of BG11 medium^44^. The flasks were placed in a 28 °C illuminated incubator under a 12 h light/12 h dark photoperiod and illuminated by fluorescent lamps on top of the flasks with a light density of 40 μE m^−2^ s^−1^.

### Experimental design

Pre-cultures of *S*. *obliquus* at exponential phase were inoculated into fresh BG11 medium with an initial cell density of 0.5–1.0 × 10^7^ cells/mL for algae growth assay. Before the inoculation of algal cells, different concentrations of the filtered suspension of test NPs were added into the growth media. Nominal CNTs concentrations were set at 2, 5, 10, 15, 40 mg·L^−1^. The nominal concentrations for nano Fe_2_O_3_ for the test in the medium were 2, 5, 10, 20, 40, 60 and 100 mg·L^−1^, and 0.8, 8, 40 and 100 mg·L^−1^ for nano MgO. In the exposure test, cultures were grown in 250 mL Erlenmeyer flasks containing 150 mL of modified BG11 medium containing different concentrations of nanoparticles. The cultivation conditions were as described in Sec. 2.2 and each treatment was carried out in triplicate.

Algae cells were grown in medium containing various NPs concentration for 7–8 days and algal growth was monitored daily. Cell density was measured by cell counting using a hemocytometer and an optical microscope (Leica, Germany). Growth inhibition (%) was calculated from difference of the specific growth rate in untreated and treated cultures at 96 h. EC_30_ and EC_50_ for algal growth in response to NPs were calculated from the growth inhibition by nonlinear regression using the cumulative distribution function pnorm of the statistical software SPSS 16.0. The 95% confidence intervals for the EC_30_ and EC_50_ values were calculated using bootstrap resampling.

### Determination of biochemical compounds and antioxidant enzyme activities

Chlorophyll determination was performed by 100% methanol extraction and the absorbance of supernatant was measured at the wavelengths of 653 nm and 666 nm^45^. Cells at 96 h were harvested and analyzed for total sugar, soluble protein and antioxidant enzyme activity analysis. Total sugar content were measured by phenol-sulfuric acid method^46^. Total soluble protein contents were determined by Bicinchoninic acid method using the total protein assay kit (Nanjing Jiancheng Biology Engineering Institute, China), and the results were expressed as micrograms of protein per 10^7^ cells (μg/10^7^cell). The CAT and POD activities were measured by assay kits (Nanjing Jiancheng Biology Engineering Institute, China). The SOD activity was determined according to the method of Mishra^47^. The activity of SOD was defined as the quantity of SOD required to inhibit 50% the photochemical reduction of nitroblue of tetrazolium.

Neutral lipid content was determined via monitoring the nile red fluorescence intensity of the algae samples as described previously^48^. Total lipid was exacted using methanol: chloroform mixture (2:1 v/v) according to Gao *et al*.^49^ and the content was expressed as percentage per dry cell weight (DCW). The lipid composition was determined as fatty acid methyl esters through direct transesterification with potassium hydroxide (KOH) in methanol. The crude lipid sample was analyzed using a gas chromatograph (Thermo Trace GC ULTRA) equipped with a flame ionization detector (FID) as described previously^49^.

### Measurement of MDA and hydrogen peroxide

MDA was measured spectrophotometrically based on the thiobarbituric acid method^50^. Algae samples of 50 mL after 48 h exposure were harvested and homogenized in an ice bath by ultrasonication as described above, then resuspended in 4 mL 80% ethanol. The supernatants were collected and the MDA content was determined by a commercial kit (Nanjing Jiancheng Biology Engineering Institute, China) following the manufacturer’s instruction. MDA content was expressed as nmol MDA/mg protein. The amount of hydrogen peroxide was determined using the TiCl_4_ method based on the reaction between hydrogen peroxide and TiCl_4_ that forms colored Ti complexes. The absorbance was measured at 410 nm. The H_2_O_2_ content was expressed as mmol H_2_O_2_/mg protein.

### Photocatalytic Degradation of methylene blue (MB)

Methylene blue is a widely used model dye to assess the photo catalytic potential of nanoparticles. It is also used to confirm intended oxidative stress caused by the photocatalyst arises in a microalgae cultivation system^14^. Typically 10 mg of tested NPs (α-Fe_2_O_3_ and MgO) were added to 100 mL of methylene blue dye solution (10 mg·L^−1^). The mixtures were well mixed by magnetically stirring for 30 min to equilibrate the working solution before illumination. The suspensions were then exposed to illumination and the absorbance at 668 nm was monitored at a 60 min interval. Percentage of dye degradation was estimated by the following formula:

$${\rm{Decolourization}}\,( \% )=({{\rm{OD}}}_{{\rm{i}}}-{\rm{OD}}){/\mathrm{OD}}_{{\rm{i}}}\,\ast \,100,$$

where OD_i_ is the initial concentration of dye solution and OD is the concentration of dye solution after photocatalytic degradation.

### Measurement of photosynthetic parameters

The chlorophyll fluorescence parameters were measured by using a multi-wavelength phytoplankton pulse-amplitude-modulated fluorometer (Phyto-PAM, Walz, Germany)^51^. The maximal efficiency of photosystem II photochemistry, F_v_/F_m_, was measured after dark adaption of the samples for 10 min. Rapid light curves (RLCs) were performed according to a pre-installed software routine. rETR_max_ (the relative maximum Electron Transport Rate) was calculated from the RLCs^52^.

### Scanning electron microscopic (SEM) imaging

After exposure to NPs for 48 h, the surface of the algal cells was observed by Field emission SEM (Hitachi S-4800, Japan). Aliquots of 10 mL algal suspensions under treatments with 40 mg·L^−1^ CNTs, 60 mg·L^−1^ nano Fe_2_O_3_, and 40 mg·L^−1^ nano MgO were withdrawn and cells were harvested at 1000 g for 3 min. The pellets were then chemically fixed for ~24 h using 4% (w/v) glutaraldehyde. Then the samples were subjected to a dehydration procedure by graded series of ethanol before air-drying. Finally, the sample films were coated with gold and loaded for SEM analysis. Each sample was analyzed for at least five view fields at different magnifications.

### Measurement of Fe^3+^ concentration in the culture media

An aliquot of 2 mL culture was sampled from treatments exposed to different nano Fe_2_O_3_ concentrations for 3 days, then filtering through a 0.45 μm membrane. The filtrate was collected for analyzing Fe^3+^ concentration by an inductively coupled plasma - mass spectrometer (ICP-MS, Agilent 710).

### Statistical analysis

SPSS PASW Statistics 16 software was used for all statistical analyses. The mean values, confidence intervals, and standard deviation values for all treatments (triplicates) were calculated. Data from three replicates (*n* = 3) were analyzed using one-way ANOVA, and *p* < 0.05 was considered statistically significant.

### Data availability statement

The datasets generated during and/or analysed during the current study are available from the corresponding author on reasonable request.

## Electronic supplementary material

Supplementary data
