# Supplementary material for: Improvement on lipid production by Scenedesmus obliquus triggered by low dose exposure to nanoparticles
Source: Sci Rep. 2017 Nov 14;7:15526. doi: 10.1038/s41598-017-15667-0 (PMC5686080; doi:10.1038/s41598-017-15667-0)
Supplement: Supplementary file 1 — Supplementary data [file 41598_2017_15667_MOESM1_ESM.doc]

**Improvement on lipid production by *Scenedesmus obliquus* triggered by low dose exposure to nanoparticles**

Meilin He, Yongquan Yan, Feng Pei, Mingzhu Wu, Temesgen Gebreluel, Shanmei Zou, Changhai Wang***

Jiangsu Key Laboratory of Marine Biology, College of Resources and Environmental Science, Nanjing Agricultural University, Nanjing 210095, China

* Corresponding author. Tel.: 0086 25 84396680; E-mail: chwang@njau.edu.cn

Address: NO. 1 Weigang, Nanjing, 210095, China


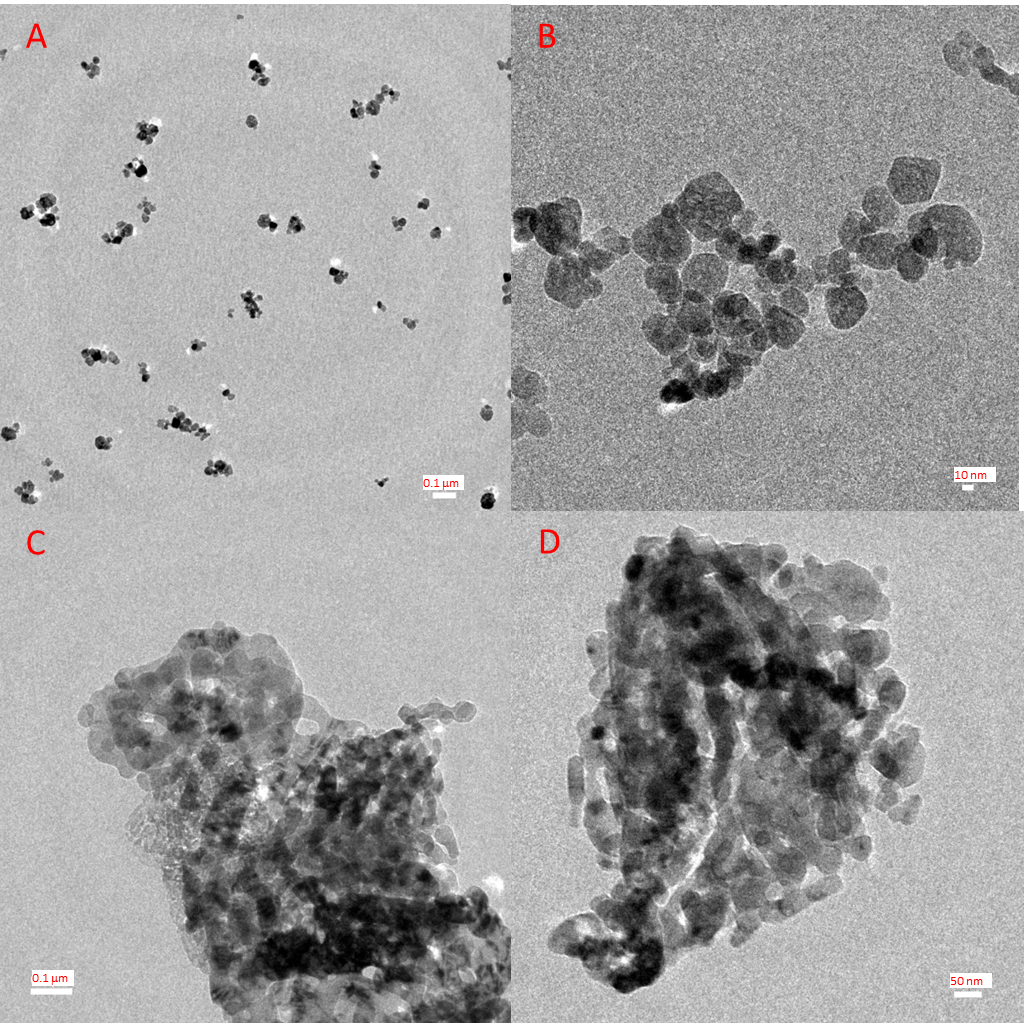


Fig. S1 TEM image of (A) and (B) nano Fe_2_O_3_, (C) and (D) nano MgO

Table S1 Comparison of various biochemical compounds content per algal cells exposed to CNTs, nano Fe_2_O_3_ and nano MgO

|  | NPs (mg·L^-1^) | Chlorophyll (μg/10^8^ cells) | Protein (mg/L) | Soluble sugars (mg/L) |
| --- | --- | --- | --- | --- |
| CNTs | 0 | 8.12±0.35 | 8.77±0.82 | 12.17±0.95 |
|  | 2.5 | 8.56±0.65 | 9.13±1.73 | 16.05±3.11 ^*^ |
|  | 5 | 9.28±0.23 ^*^ | 10.14±1.72 | 17.23±2.01 ^*^ |
|  | 10 | 8.94±0.65 ^*^ | 9.02±1.3 | 14.02±1.56 ^*^ |
|  | 15 | 6.41±0.61 ^*^ | 7.66±1.08 | 16.59±2.74 ^*^ |
|  | 40 | 6.40±0.30 ^*^ | 7.42±0.44 | 12.53±1.06 |
| Nano Fe_2_O_3_ | 0 | 5.47±0.11 | 7.2±0.96 | 10.5±0.55 |
|  | 2 | 6.14±0.18 ^*^ | 9.67±0.54 ^*^ | 13.78±1.07 ^*^ |
|  | 5 | 6.15±0.08 ^*^ | 8.33±1.36 ^*^ | 17.24±2.08 ^*^ |
|  | 10 | 5.88±0.52 | 10.46±1.53 ^*^ | 18.59±0.81 ^*^ |
|  | 20 | 5.13±0.36 | 9.35±1.08 ^*^ | 15.03±1.17 ^*^ |
|  | 40 | 3.84±0.09 ^*^ | 7.69±0.13 | 14.11±1.56 ^*^ |
|  | 60 | 3.76±0.10 ^*^ | 7.51±0.94 | 15.80±2.03 ^*^ |
|  | 100 | 3.99±0.17 ^*^ | 7.05±0.34 | 14.18±2.48 ^*^ |
| Nano MgO | 0 | 15.62±1.25 | 10.31±0.34 | 11.46±0.14 |
|  | 0.8 | 5.38±0.74 ^*^ | 10.08±0.17 ^*^ | 11.61±0.07 |
|  | 8 | 3.75±0.61 ^*^ | 9.45±0.33 ^*^ | 9.18±0.16 ^*^ |
|  | 40 | 5.14±0.73 ^*^ | 6.59±0.33 ^*^ | 13.88±0.28 ^*^ |
|  | 100 | 9.36±0.34 ^*^ | 4.78±0.42 ^*^ | 7.36±0.34 ^*^ |

Data are represented as mean ± SD from triplicate samples (*n=*3). Significant difference (*p*<0.05) between treatments was indicated by asterisks.
